# Anterior Chamber Angle and Volume Do Not Change after Myopic Laser-Assisted In Situ Keratomileusis in Young Patients

**DOI:** 10.1155/2018/8646275

**Published:** 2018-12-10

**Authors:** Ertuğrul Tan Yassa, Cihan Ünlü

**Affiliations:** ^1^MD, FEBO, Asya Eye Medical Centre, Ophthalmology Department, Istanbul 34310, Turkey; ^2^MD, Asya Eye Medical Centre, Ophthalmology Department, Istanbul 34310, Turkey

## Abstract

**Purpose:**

We aimed to investigate whether anterior chamber angle, depth, and volume change after myopic laser-assisted in situ keratomileusis (LASIK) in young patients.

**Methods:**

This retrospective study included 29 eyes of 29 patients (15 females and 14 males) between the ages of 18 and 39 years who underwent LASIK for the treatment of myopia. In addition to complete ophthalmic examination, anterior chamber angle (ACA), central anterior chamber depth (ACD), and anterior chamber volume (ACV) were measured by using a Sirius rotating Scheimpflug camera combined with Placido disc corneal topography (CSO, Florence, Italy). Preoperative and postoperative values were compared using paired *t-*tests. Linear regression was used to evaluate the relationship between changes in refraction and changes in ACA, ACD, and ACV as well as between attempted maximum ablation depth and changes in ACA, ACD, and ACV.

**Results:**

The mean age of the study population was 25.5 ± 6.2 years. The mean preoperative and postoperative spherical equivalent values were −3.30 ± 1.92 D and −0.18 ± 0.29 D, respectively. ACV and ACA were not significantly changed after surgery; however, central ACD was significantly decreased (preoperatively = 3.366 ± 0.316 mm vs postoperatively = 3.3 ± 0.298 mm) with a mean difference of 0.066 ± 1.121 mm. There were no significant relationships between changes in refraction and anterior segment dimensions or between attempted maximum ablation depth and anterior segment dimensions.

**Conclusion:**

Measurements with Sirius showed that ACA and ACV did not change; however, central ACD significantly decreased after myopic LASIK in young patients, indicating that the preoperative central ACD value should not be used interchangeably with the postoperative central ACD value.

## 1. Introduction

Accurate assessment of anterior chamber dimensions has several clinical applications. First, accurate measurement of anterior chamber angle (ACA) and anterior chamber depth (ACD) is crucial for the diagnosis and follow-up of angle-closure glaucoma. Additionally, ACD and anterior chamber volume (ACV) values are considered the primary determinants for developing angle-closure [1]. Second, ACD is an important parameter in the precise calculation of intraocular lens (IOL) power along with axial eye length and corneal power measurements, in converting toric IOL cylindrical power from IOL plane to corneal plane [2, 3], and in selection of IOL size and patients for phakic IOL implantation [4, 5].

Based on these reasons, several studies have been conducted to evaluate ACD in patients who had undergone laser-assisted in situ keratomileusis (LASIK) for the treatment of myopia. Nishimura et al. [6] found that central ACD was not changed after LASIK in contrast with several studies reporting that central ACD significantly decreased [7–9]. ACV was investigated in two studies, one of which found ACV to be constant [6] and the other one of which found it to be decreased after surgery [9]. Furthermore, to the best of the authors' knowledge, there is no study to date reporting ACA changes following LASIK for the treatment of myopia. Thus, in this study, we investigated ACA, ACD, and ACV changes in patients who had undergone myopic excimer laser surgery using the Sirius rotating Scheimpflug camera combined with Placido disc corneal topography (CSO, Florence, Italy), which are of clinical importance.

## 2. Methods

The Medical Ethics Committee of the Bakirköy Dr. Sadi Konuk Training and Research Hospital of the Ministry of Health University approved the present study; the study also adheres to the principles of the Declaration of Helsinki. We retrospectively studied the medical records of 29 eyes of 29 patients (15 females and 14 males) who had undergone LASIK for the treatment of myopia and/or myopic astigmatism at the Asya Eye Medical Centre between June 2017 and May 2018. Patients who were included in the study were candidates for refractive surgery between the ages of 18 to 39 years who had exhibited stable refraction for at least 12 months. The exclusion criteria for the study included retreatment, intraoperative and/or postoperative complications, ocular diseases other than myopia and/or myopic astigmatism, systemic diseases, autoimmune diseases, breastfeeding, and pregnancy.

Preoperative and postoperative cycloplegic refraction, air-puff tonometry, anterior segment evaluation, and dilated fundus examination were performed on all patients. We measured ACA, central ACD, and ACV using a Sirius running Phoenix software (version 3.4.0.73), both before surgery and 1 to 3 months after surgery. The Sirius uses 475 nm wavelength blue LED flash illumination and derives these parameters solely from its Scheimpflug camera. Two experienced nurses conducted all measurements at the same time of the day, between 10:00 and 12:00 hours, under dim light conditions. The Sirius provides both nasal and temporal anterior chamber angles. The Sirius device measures central ACD from the endothelial side of the cornea to the anterior surface of the crystalline lens. In refractive surgery, a Moria 2 microkeratome (Moria, Antony, France) with a 90 *µ*m head was used to create superior hinged flaps. Laser ablation was performed using a MEL 90 excimer laser (Carl Zeiss Meditec, Jena, Germany). The optical zone of treatment was chosen as 6 mm (in 3 patients) or 6.5 mm (in 26 patients) according to the patient's refractive status, corneal topography findings, and central corneal thickness.

We performed statistical analysis using Prism 7 software (Graphpad Software, Inc.). A *P* value less than 0.05 was considered to be statistically significant. The normality of the data was investigated via the D'Agostino-Pearson normality test, and only one eye of each patient was included to prevent intercorrelation bias. We compared preoperative and postoperative measurements via two-tailed paired *t-*tests. Linear regression was used to assess the relationship between the refractive change induced by surgery (ΔSE) and nasal and temporal ACA, ACD, and ACV changes as well as between the attempted maximum ablation depth and nasal and temporal ACA, ACD, and ACV changes.

## 3. Results

The mean age of our study population was 25.5 ± 6.2 years (range, 18 to 39 years). Mean preoperative and postoperative spherical equivalent (SE) values were −3.30 ± 1.92 D and −0.18 ± 0.29 D, respectively. Mean preoperative AL (24.68 ± 1.15 mm) was significantly decreased after surgery (postoperative AL = 24.62 ± 1.12 mm, mean difference (ΔAL) = 0.0579 ± 0.0521 mm (58 *µ*m), *P* < 0.001). Mean attempted maximum ablation depth was 64.31 ± 26.15 *µ*m. Preoperative and postoperative nasal and temporal ACA, central ACD, and ACV measurements all passed the normality test (*P* > 0.05).  Table 1 shows preoperative and postoperative nasal and temporal ACAs and central ACD and ACV values. There were no significant differences between preoperative nasal and temporal ACAs and postoperative nasal and temporal ACAs (*P* > 0.05) or between preoperative ACV and postoperative ACV. The postoperative central ACD was significantly lower than the preoperative central ACD (*P* < 0.05). Linear regression did not show a significant relationship between ΔSE and nasal and temporal ACA, ACD, and ACV changes or between attempted maximum ablation depth and nasal and temporal ACA, ACD, and ACV changes (*P* > 0.05).

## 4. Discussion

The Sirius device is one of the most recently introduced anterior segment evaluation systems, and the agreement of its anterior segment measurements with previous Scheimpflug camera devices has been shown in several studies [10–12]. To the best of the authors' knowledge, this is the first study which evaluated changes in ACA after myopic LASIK and revealed that nasal and temporal ACAs do not significantly change after myopic LASIK. Zhou et al. [13] showed that, in hyperopic LASIK patients, ACA did not change after treatment. Turkish researchers Bayhan et al. [14] found a lower mean ACA nasally 44.26 ± 8.13° and temporally 45.75 ± 6.76° than our findings using a Sirius device in a cohort of 80 healthy subjects (mean SE of −0.44 ± 1.2 D).

The present study shows that central ACD is significantly decreased after myopic LASIK treatment. Similarly, Cairns et al. [7] and Hashemi and Mehravaran [8] reported a significant decrease in ACD but Nishimura et al. [6] did not find a significant change in central ACD values after LASIK in their first study. However, in a later study, they described central ACD as significantly decreased only in young patients (mean central ACD of 3.26 ± 0.22 mm preoperatively vs mean central ACD of 3.22 ± 0.22 mm postoperatively, *P* < 0.001, mean age = 30.4 ± 4.2 years) in contrast with results for older patients (mean central ACD of 3.15 ± 0.34 mm preoperatively vs mean central ACD of 3.15 ± 0.33 mm postoperatively, *P*=0.514, mean age = 45.8 ± 5.1 years) [9]. Similar to the latter study, the mean age was 25.5 ± 6.2 years in our study. Kazancı et al. [15] found central ACD was 3.01 ± 0.33 mm in 107 Turkish subjects with low refractive errors (myopia and hyperopia lower than 3D and astigmatism lower than 2D) using a Sirius device, a result that is lower than ours.

There is still no agreement on the definite alterations in the posterior corneal surface after myopic refractive surgery. Studies have shown that the posterior cornea is stable [16, 17] or moves only slightly forward [18] after LASIK. Most recently, Chan et al. [18] stated that the posterior cornea moves slightly forward in LASIK and photorefractive keratectomy patients (4.88 ± 0.47 *µ*m and 3.67 ± 0.48 *µ*m, respectively) in a study utilising swept-source optical coherence tomography. Based on these observations, a decrease of 0.066 mm in the central ACD as found in this study cannot be explained solely without forward movement of the human crystalline lens. Tsorbatzoglou et al. [19] found that accommodation-induced central ACD decreases were 0.08 ± 0.06 mm in patients younger than 30 years old and 0.064 ± 0.087 mm in patients between 31 and 40 years of age. Additionally, improved accommodation parameters were reported after implantation of phakic posterior chamber implantable contact lens (ICL) or after femtosecond laser-assisted small lenticule extraction (SMILE) for myopia in studies by Fu et al. [20] and Zheng et al. [21], respectively. Combining all of the findings noted above, we hypothesised that LASIK treatment might similarly improve accommodation when performed on young myopic patients, resulting in more accommodative response postoperatively than preoperatively to Sirius's fixation target despite setting it at optical infinity. The issue of persistent accommodation against the fixation target of Scheimpflug devices at optical infinity has also been discussed by Nishimura et al. [9] and Cairns et al. [7] in explaining central ACD changes found in their studies.

According to the results of this study, ACV does not change significantly after myopic LASIK. A decrease in central ACD concurrently with stable ACA and ACV measurements might seem paradoxical at first glance, but Dubbelman et al. [22] found that the anterior surface of the crystalline lens becomes more hyperbolic and curved, shifting towards the cornea centrally, while becoming flatter peripherally during accommodation. We speculate that these regional disparities of the anterior lens surface at the centre and periphery when our study population looked at the Sirius fixation target, combined with forward displacement of the posterior cornea after LASIK, might explain why ACV and ACA did not change significantly while the central ACD decreased after surgery. In contrast to our study, Nishimura et al. [9] found that ACV significantly decreased after surgery in young patients. The disparity might be related to differences in the mean optical zone of treatment (5.18 ± 0.76 mm in Nishimura et al.'s [9] younger group vs 6.45 ± 0.16 mm in this study) and preoperative SE (−5.95 ± 2.13 D in Nishimura et al.'s [9] younger group vs −3.30 ± 1.92 D in this study) in these studies. ACV was described as 160.1 ± 33.1 *µ*L using a Sirius in Turkish subjects with low refractive errors (myopia and hyperopia lower than 3D, astigmatism lower than 2D), a result that is lower than ours [15].

The limitations of the present study are as follows. First, a single measurement was performed on all patients because repeatability of ACA [23, 24], central ACD [10–12, 23, 25], and ACV [25] measurements of the Sirius in unoperated eyes and those after refractive surgery [25] has been confirmed by other studies. Second, we did not verify accommodative alterations because of the retrospective nature of this study. Thus, further prospective studies with and without cycloplegia are still needed to clarify the effects of accommodation on the dimensions of the anterior segment before and after surgery. Third, we measured only central ACD in the present study because the software used with the Sirius device does not automatically provide ACD at other locations (e.g., nasal, temporal, inferior, and superior locations). Finally, our follow-up time is short, but similar studies had similar follow-up times, for instance, one month [6, 9, 13], six weeks [8], or 8.3 ± 4.0 weeks [7].

In conclusion, according to the results of this study, central ACD significantly decreased after myopic LASIK; however, ACA and ACV did not. These results may possibly relate to a combination of region-dependent shape changes happening at the lens anterior surface during fixation of the patient's eye on the Sirius fixation target and posterior corneal changes happening after surgery. These results are clinical relevant: first, because the anterior segment dimensions of this myopic study cohort were still greater postoperatively than those of healthy patients with the same nationality [14, 15], patients who had undergone myopic LASIK surgery do not seem to be at higher risk for angle-closure than the normal population. Nevertheless, clinicians should still exclude patients with high risk of angle-closure from LASIK to increase the safety and efficacy of the surgery. Second, in this study, the central ACD decreased from 3.336 mm to 3.3 mm after surgery, so previous myopic LASIK history does not seem to influence patients' candidacy for phakic IOL treatment (e.g., for the treatment of presbyopia) because surgeons consider patients with more than 2.8 mm of central ACD as candidates for this treatment. Finally, the preoperative central ACD value should not be used interchangeably with the postoperative ACD value.

## Figures and Tables

**Table 1:** Preoperative and postoperative ACA, ACD, and ACV values.

Anterior segment parameter	Preoperative	Postoperative	Mean difference	*P*	*n*
nACA (°)	43.04 ± 9.84	42.25 ± 11.23	−0.79 ±7.71	0.59	28
tACA (°)	50.79 ± 5.92	49.57 ± 5.34	−1.21 ± 5.68	0.27	28
ACD (mm)	3.366 ± 0.316	3.3 ± 0.298	−0.066 ± 0.121	0.007^*∗*^	29
ACV (*µ*L)	182.7 ± 33.91	183.3 ± 32.94	0.586 ± 16.51	0.85	29

nACA: nasal anterior chamber angle; tACA: temporal anterior chamber angle; ACD: anterior chamber depth; ACV: anterior chamber volume. ^*∗*^*P* < 0.05 was statistically significant; values are presented as mean difference ± standard deviation.

## Data Availability

The data used to support the findings of this study are available from the corresponding author upon request.
